# Femoral Reconstruction Using a Push-Through Prosthesis for Massive Femoral Bone Loss: A Retrospective Case Series and Review of the Literature

**DOI:** 10.7759/cureus.114612

**Published:** 2026-08-16

**Authors:** David Mayorga-Naranjo, Amparo Ortega-Yago, Ignacio Baixauli-García, Francisco Argüelles-Linares, Francisco Baixauli-García, José Baeza-Oliete

**Affiliations:** 1 Department of Orthopedic Surgery and Traumatology, Hospital Universitari i Politècnic La Fe, Valencia, ESP

**Keywords:** femoral reconstruction, limb salvage surgery, massive femoral bone loss, megaprosthesis, push-through prosthesis

## Abstract

Massive femoral bone loss remains one of the greatest challenges in limb salvage and revision orthopedic surgery. The push-through prosthesis was developed as an alternative to total femoral replacement, allowing preservation of the remaining femoral cortex and its associated muscular attachments; however, clinical evidence regarding its use remains limited. We retrospectively reviewed all patients who underwent femoral reconstruction using a push-through prosthesis at a tertiary referral center between 2006 and 2025. Demographic characteristics, surgical indications, previous procedures, postoperative complications, implant survival, and functional outcomes were analyzed, and the current literature was reviewed. Five patients (mean age, 63.4 years) with massive femoral bone loss following complex reconstructive procedures were included. Surgical indications comprised periprosthetic joint infection (n = 2), complex femoral nonunion (n = 1), mechanical failure of a megaprosthesis (n = 1), and failure of revision total hip arthroplasty (n = 1). At a mean follow-up of more than five years, no cases of aseptic loosening, periprosthetic fracture, prosthetic dislocation, or recurrent infection were observed. No patient required revision surgery, resulting in 100% implant and limb survival. All patients remained ambulatory at final follow-up, although most required walking aids. These findings suggest that the push-through prosthesis is a valuable limb-salvage option for carefully selected patients with massive femoral bone loss when conventional reconstructive techniques are no longer feasible, providing durable implant survival, limb preservation, and satisfactory functional outcomes. However, these findings should be interpreted with caution given the small sample size and the absence of standardized functional outcome measures, highlighting the need for larger multicenter studies.

## Introduction

Reconstruction of massive femoral bone loss remains one of the most demanding challenges in contemporary reconstructive orthopedic surgery. This clinical scenario may arise following multiple revision arthroplasties, chronic periprosthetic joint infection, complex nonunion, unreconstructable periprosthetic fractures, or tumor resection. In such cases, extensive bone loss combined with compromised soft tissues markedly limits the available reconstructive options [1-4].

When bone loss involves a substantial portion of the femur, conventional reconstruction techniques-including diaphyseal fixation revision stems, structural allografts, and allograft-prosthetic composites-often yield unpredictable outcomes and are associated with high complication rates. Consequently, total femoral replacement (TFR) has traditionally been considered the salvage procedure of choice, particularly when insufficient host bone remains to achieve stable fixation with standard revision implants [5-7].

Although TFR enables limb preservation in patients with catastrophic femoral bone defects, its use, particularly in nononcological indications, has consistently been associated with high rates of mechanical and septic complications. Reported failure rates range from 30% to 60%, with instability, infection, aseptic loosening, periprosthetic fracture, and repeated revision surgery representing the most frequent causes of implant failure [7-10].

To preserve the remaining femoral cortex and maintain the native muscular attachments, the push-through prosthesis (Waldemar LINK®, Hamburg, Germany) was developed as an alternative to conventional TFR. Unlike standard TFR, this modular system preserves a residual cortical femoral shell through which an intramedullary stem is inserted. By maintaining part of the native femoral cortex and the surrounding soft-tissue envelope, this reconstructive strategy aims to improve biomechanical stability, preserve the abductor mechanism, and facilitate potential future revision procedures. Nevertheless, the clinical evidence supporting these theoretical advantages remains scarce [11-14].

To date, the published experience with this technique is extremely limited and is largely confined to the institution where the implant was originally developed. Existing reports comprise small, heterogeneous series including both oncological and nononcological indications, providing only limited information regarding implant survival, complication rates, and functional outcomes, particularly in patients undergoing complex revision surgery or reconstruction following periprosthetic joint infection [14,15]. Furthermore, no previous study has specifically evaluated the performance of this implant in a consecutive cohort from an independent tertiary referral center specializing in complex revision arthroplasty and bone and joint infection. Consequently, the generalizability of the currently available evidence remains uncertain.

The primary aim of the present study was to evaluate the clinical and functional outcomes of a consecutive series of patients treated with femoral reconstruction using a push-through prosthesis at a tertiary referral center. Specifically, we assessed postoperative complications, implant survival, and limb survival. As a secondary objective, we performed a comprehensive review of the available literature to place our findings in context and to better define the current role of this technique within the armamentarium of limb salvage reconstruction for massive femoral bone loss. To the best of our knowledge, this represents one of the few reported clinical series from an independent tertiary referral center, providing additional evidence on the use of the push-through prosthesis in predominantly nononcological complex reconstructive cases.

## Case presentation

Between January 2006 and April 2025, five consecutive patients underwent femoral reconstruction using a push-through prosthesis at our tertiary referral center and national reference institution for the management of complex osteoarticular infection and revision reconstructive surgery. Patients were identified through a retrospective review of the institutional surgical database and electronic medical records. This retrospective case series includes all consecutive adult patients who underwent femoral reconstruction using a push-through prosthesis during the study period and met the predefined inclusion criteria, thereby minimizing selection bias.

The study was conducted in accordance with the principles of the Declaration of Helsinki and current national regulations governing biomedical research. Owing to its retrospective design and the exclusive use of anonymized clinical data, the requirement for informed consent was waived according to institutional policy.

Patient selection

The inclusion and exclusion criteria are summarized in Table 1.

**Table 1:** Inclusion and exclusion criteria

Type	Criterion
Inclusion	Age ≥18 years
	Femoral reconstruction using a push-through prosthesis
	Massive femoral bone loss
	Surgery performed between January 2006 and April 2025
	Minimum follow-up of 12 months
Exclusion	Conventional total femoral replacement
	Segmental megaprosthesis without a push-through prosthesis
	Incomplete clinical data preventing analysis

Surgical indications

The indication for reconstruction with a push-through prosthesis was established on an individual basis following multidisciplinary assessment by the institutional bone and joint infection board. In every case, patients presented with extensive femoral bone loss after multiple previous surgical procedures, precluding the use of standard revision implants. Treatment decisions were reached by a multidisciplinary team comprising orthopedic surgeons specialized in complex reconstruction, infectious disease physicians, microbiologists, musculoskeletal radiologists, and plastic surgeons, when required. Bone stock, soft-tissue quality, the presence or absence of active infection, patient comorbidities, and expected postoperative function were considered before selecting the reconstructive strategy.

Surgical technique

All procedures were performed by surgeons experienced in complex reconstructive surgery using the modular push-through prosthetic system (Waldemar LINK®, Hamburg, Germany). Following complete removal of previous fixation devices or prosthetic components, meticulous debridement of all devitalized tissue was undertaken. Whenever infection was suspected, multiple tissue samples were obtained for microbiological and histopathological analysis before antibiotic administration. Whenever feasible, the remaining cortical femoral shell was preserved, providing the biological foundation for reconstruction.

The femoral canal was then prepared, and the modular intramedullary component of the push-through prosthesis was inserted through the residual cortical cylinder. Implant reconstruction was performed using a modular assembly tailored to the extent of femoral bone loss and each patient's individual anatomical requirements. The proximal and distal articulations were reconstructed according to the remaining bone stock and the existing implants, with the objective of restoring limb length, mechanical stability, and joint function while preserving as much native soft-tissue attachment as possible.

In patients with previous infection, postoperative antimicrobial therapy was determined jointly with the Infectious Diseases Department according to microbiological culture results and antimicrobial susceptibility testing.

Follow-up

Postoperative rehabilitation was individualized according to bone stock, soft-tissue reconstruction, and intraoperative implant stability. Early mobilization under physiotherapist supervision was encouraged, with progression of weight-bearing determined according to the treating surgeon's assessment and serial radiographic evaluation.

Patients underwent a standardized follow-up protocol including clinical and radiographic assessments at two weeks, one month, three months, and six months after surgery, followed by annual reviews thereafter. At each visit, patients were evaluated for clinical signs of infection, implant stability, mechanical complications, soft-tissue healing, and functional recovery through physical examination, laboratory investigations, and radiographic assessment. Patients treated for periprosthetic joint infection were followed jointly by the Orthopedic Surgery and Infectious Diseases teams. Demographic, clinical, surgical, microbiological, and postoperative data were retrospectively collected from the institutional electronic medical records. The mean follow-up was 6.4 years (median, 7 years; range, 5-7 years). Implant survival was defined as the interval between implantation of the push-through prosthesis and revision or removal of the implant for any cause.

Patient outcomes

Five consecutive patients underwent femoral reconstruction using a push-through prosthesis. The demographic and clinical characteristics of the study cohort are summarized in Table 2. All procedures were performed as limb salvage surgery following failure of previous complex reconstructive procedures and were undertaken by the same senior reconstructive team at our institution.

**Table 2:** Demographic and clinical characteristics of the study cohort BMI: body mass index

Patient	Age (years)	Sex	BMI (kg/m²)	Comorbidities	Surgical indication	Previous operations (n)	Microbiology	Follow-up (years)	Walking aid at final follow-up	Complications
1	45	Male	31.3	None	Sequelae of femoral osteomyelitis	4	None	5	One crutch	None
2	84	Female	24.0	Parkinson's disease, hypothyroidism	Periprosthetic joint infection	3	Corynebacterium mucifaciens	7	Walker	None
3	80	Female	17.9	Osteoporosis, glaucoma	Complex femoral nonunion	3	Staphylococcus epidermidis	7	Walker	None
4	48	Female	42.0	None	Mechanical failure of a megaprosthesis following osteosarcoma	2	None	7	None	None
5	60	Male	19.4	Rheumatoid arthritis (chronic corticosteroid therapy)	Failed revision total hip arthroplasty	3	Staphylococcus capitis	6	Crutches	Thrombocytopenia and toxicoderma; death due to disseminated tuberculosis (unrelated to the implant)

**Figure 1:**
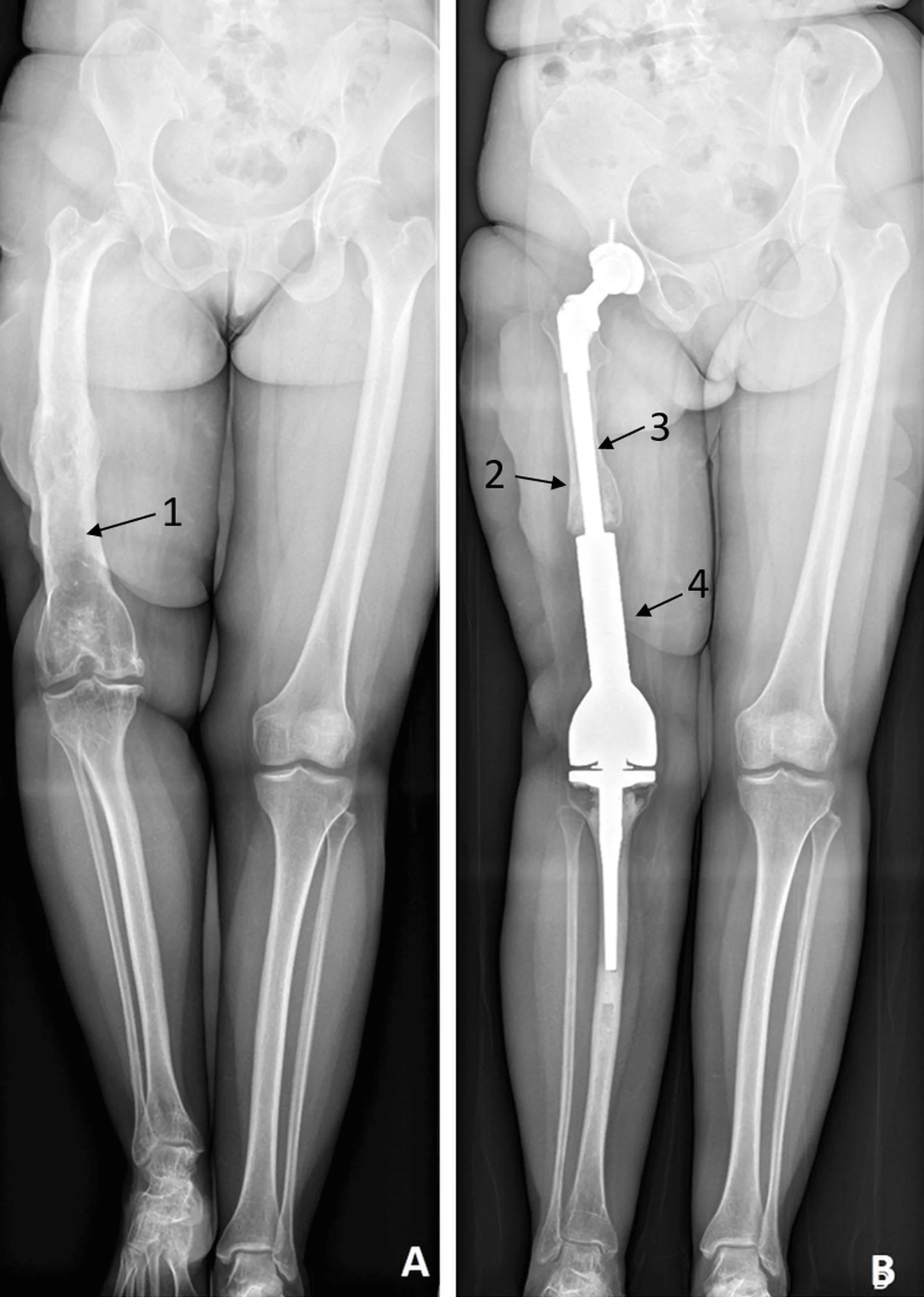
Representative anteroposterior radiographs of a patient treated with a push-through prosthesis for the reconstruction of massive femoral bone loss secondary to chronic osteomyelitis (A) Preoperative radiograph showing extensive femoral bone loss (1). (B) Postoperative radiograph following reconstruction with a push-through prosthesis demonstrating preservation of the residual femoral cortical shell (2), the intramedullary push-through stem traversing the preserved cortical cylinder (3), and the modular distal femoral reconstruction (4)

The mean age at surgery was 63.4 years (range, 45-84 years). Four (80%) were female patients, and one (20%) was male patients. All patients presented with extensive femoral bone loss following multiple previous reconstructive procedures, with a median of three previous surgical interventions before definitive implantation of the push-through prosthesis. Representative radiographs of a patient treated with a push-through prosthesis are shown in Figure 1.

The indications for reconstruction included chronic periprosthetic joint infection with extensive femoral bone loss after multiple revision procedures (n = 2), complex femoral nonunion following repeated failed internal fixation (n = 1), mechanical failure of a tumor megaprosthesis implanted for femoral osteosarcoma (n = 1), and catastrophic failure of a revision total hip arthroplasty with intrapelvic migration of the femoral component (n = 1). In every patient, conventional reconstructive techniques were considered unsuitable because of the magnitude of bone loss and the complexity of the underlying clinical condition.

All reconstructions were performed using the modular push-through prosthetic system (Waldemar LINK®, Hamburg, Germany). In the two patients with active periprosthetic joint infection, extensive surgical debridement was undertaken, and multiple tissue specimens were obtained for microbiological analysis before definitive implantation. The isolated microorganisms were *Staphylococcus epidermidis* and *Corynebacterium mucifaciens*, and both patients received culture-directed antimicrobial therapy. No bacterial growth was identified in the remaining three patients, confirming aseptic failure.

During follow-up, no intraoperative complications or major implant-related mechanical failures were observed. Specifically, there were no cases of aseptic loosening, periprosthetic fracture, modular component failure, or prosthetic dislocation. Furthermore, none of the patients treated for periprosthetic joint infection developed clinical or microbiological recurrence during follow-up. No patient required revision surgery related to the push-through prosthesis, and no amputations or hip disarticulations were performed, resulting in 100% implant survival and 100% limb survival at final follow-up.

From a functional standpoint, all patients remained ambulatory. At the latest follow-up, one patient walked independently, one required a single crutch, two used a walker for routine ambulation, and one required bilateral crutches. Overall, all patients achieved a stable, functional limb capable of supporting activities of daily living, although varying degrees of dependence on walking aids persisted. One patient died during follow-up from disseminated tuberculosis, which was unrelated to the orthopedic procedure or the implanted prosthesis.

## Discussion

The management of massive femoral bone loss remains one of the greatest challenges in reconstructive orthopedic surgery. Patients requiring these procedures have typically undergone multiple previous operations and often present with compromised soft tissues, chronic periprosthetic joint infection, or failure of previous reconstructive attempts. Consequently, treatment options are limited and are frequently associated with high complication and reoperation rates. In this setting, the primary objective of reconstruction is not necessarily to restore normal anatomy or function but rather to preserve a stable, painless, and functional limb while avoiding ablative procedures such as amputation or hip disarticulation [1-4].

The principal finding of the present study is that femoral reconstruction using a push-through prosthesis achieved complete implant and limb survival, with no cases of mechanical failure, recurrent infection, or revision surgery during a mean follow-up exceeding five years. Although the cohort is small, these findings are noteworthy because all patients had undergone multiple previous reconstructive procedures and presented with severe femoral bone loss in whom conventional reconstructive strategies had been exhausted.

TFR remains the most widely used salvage procedure for extensive femoral bone defects. However, several studies have demonstrated that outcomes are substantially less favorable in nononcological indications than in tumor surgery. Reported overall complication rates range from 30% to 70%, with instability, aseptic loosening, periprosthetic fracture, reinfection, and repeated revision surgery representing the most common causes of failure [5-9]. These poorer outcomes are likely related to chronic infection, multiple previous procedures, compromised soft tissues, and the greater medical complexity of patients undergoing revision surgery.

The push-through prosthesis was specifically designed to preserve the remaining femoral cortical shell and, whenever possible, the proximal muscular attachments. In contrast to conventional TFR, this reconstructive philosophy maintains part of the native biological envelope, which may improve load transfer, enhance implant stability, preserve the abductor mechanism, and potentially reduce the risk of instability. Although these theoretical biomechanical advantages have been widely discussed, clinical evidence remains scarce owing to the rarity of the procedure [11-14].

Appropriate patient selection is fundamental when considering reconstruction with a push-through prosthesis. This technique appears particularly suitable for carefully selected patients with massive femoral bone loss in whom preservation of a residual femoral cortical shell remains feasible, allowing maintenance of the native soft-tissue envelope and muscular attachments. Conversely, patients with complete femoral destruction or insufficient residual cortical bone may be better managed with conventional TFR. Therefore, the choice between these reconstructive strategies should be individualized according to residual bone stock, soft-tissue quality, previous surgical procedures, infection status, and the patient's expected functional demands.

To the best of our knowledge, published clinical experience with the push-through prosthesis remains limited to a small number of case series, predominantly originating from the Groningen group, who developed the implant. Gorter et al. first demonstrated that preservation of the residual femoral cortex could provide durable reconstruction with acceptable implant survival in patients with extensive femoral bone loss [14]. More recently, Bakircioglu et al. reported similarly encouraging mid-term outcomes, confirming excellent implant survival and a low incidence of mechanical complications despite the complexity of the treated cases [15]. Our findings are consistent with these reports, as no structural implant failures occurred throughout follow-up.

An important characteristic of the present series is the heterogeneity of the underlying diagnoses. Although the cohort included patients with chronic periprosthetic joint infection, complex femoral nonunion, failed revision arthroplasty, tumor-related reconstruction, and mechanical failure of a megaprosthesis, all patients shared the same reconstructive challenge: massive femoral bone loss that precluded conventional reconstructive techniques and required limb-salvage reconstruction using a push-through prosthesis. Consequently, the objective of the present study was to evaluate the reconstructive strategy itself rather than compare outcomes between different underlying pathologies, for which the sample size would not permit meaningful subgroup analyses. Unlike previous reports, which mainly include oncological reconstructions or mixed cohorts, our series consisted predominantly of nononcological cases, providing additional evidence regarding the use of this technique in complex revision arthroplasty.

Another noteworthy finding was the absence of recurrent infection among patients treated for periprosthetic joint infection. Infection remains one of the leading causes of failure following megaprosthetic reconstruction, with reported reinfection rates ranging from 15% to 35%, depending on implant location, reconstructive technique, and host-related factors [16-18]. In the present series, no recurrent infections were observed during follow-up. This favorable outcome may reflect careful patient selection, radical surgical debridement, and close collaboration with an experienced multidisciplinary bone and joint infection team. Furthermore, prolonged postoperative antimicrobial therapy was maintained until aseptic implant fixation had been confirmed. Nevertheless, given the small sample size, these findings should be interpreted with caution.

From a functional perspective, all patients remained ambulatory at final follow-up, although most required walking aids. This should not be interpreted as a limitation of the procedure but rather as an expected outcome in patients with severe bone loss, multiple previous operations, and substantial medical comorbidity. As with conventional TFR, the objective of limb salvage reconstruction is not to restore normal lower limb function but to provide a stable extremity that allows ambulation and avoids amputation. In this regard, our functional outcomes are comparable to those reported in other series of salvage megaprosthetic reconstruction, although most published studies have focused on oncological rather than infectious indications [8,19].

From a practical perspective, the widespread adoption of the push-through prosthesis may also be influenced by factors beyond clinical outcomes. Implant availability remains more limited than that of conventional megaprosthetic systems, and the procedure requires substantial experience in complex revision arthroplasty and limb salvage surgery. Careful preoperative planning and familiarity with modular reconstructive techniques are therefore essential, suggesting that this procedure is best performed in specialized tertiary referral centers. Economic considerations are also likely to influence the adoption of this technique in routine clinical practice; however, comparative cost-effectiveness studies between the push-through prosthesis and conventional TFR are currently lacking.

The strengths of this study include one of the few reported clinical series evaluating the push-through prosthesis and the predominance of nononcological indications, a patient population that remains underrepresented in the literature. In addition, the relatively long clinical follow-up provides meaningful information regarding mid-term implant durability and limb preservation.

Several limitations should be acknowledged. The retrospective design, small sample size, and absence of validated patient-reported outcome measures limit direct comparison with other published studies. However, these limitations are inherent to almost all available reports because of the exceptional rarity of this reconstructive technique. Future multicenter collaborative studies and international registries will be essential to define more precisely the indications, survivorship, complication profile, and functional outcomes associated with the push-through prosthesis.

Overall, our findings suggest that femoral reconstruction using a push-through prosthesis is a valuable limb-salvage option for carefully selected patients with massive femoral bone loss in whom conventional reconstructive techniques are no longer feasible. Nevertheless, given the complexity of these cases, this procedure should be reserved for specialized centers with extensive experience in complex revision arthroplasty and multidisciplinary management of bone and joint infection.

## Conclusions

The push-through prosthesis represents a promising limb-salvage option for selected patients with massive femoral bone loss, allowing limb preservation with high implant survival and a low complication rate in our series. Preservation of the residual femoral cortical shell constitutes the main conceptual difference between the push-through prosthesis and conventional TFR. However, given the retrospective design, small sample size, and limited evidence currently available, these findings should be interpreted with caution. Larger multicenter studies with longer follow-up and standardized functional outcome measures are required to validate these results, better define the indications for this technique, and establish its role within the reconstructive algorithm for massive femoral bone defects.
